# Comparison of T Cell Immune Responses Against SARS-CoV-2 Using QuantiFERON SARS-CoV-2 Assay Versus Peripheral Blood Mononuclear Cells Analysis by Flow Cytometry

**DOI:** 10.7759/cureus.95984

**Published:** 2025-11-03

**Authors:** Sourav Sen, Madhuri R Thakar, Prashant P Shivgunde, Shubhangi Bichare, Madhuri J Suryawanshi, Nirmalkumar Rawandale, Yogesh G Raut, Sheela V Godbole, Madhuri Kanitkar

**Affiliations:** 1 Microbiology, Maharashtra University of Health Sciences, Nashik, IND; 2 Serology/Immunology, Indian Council of Medical Research (ICMR) - National Institute of Translational Virology and AIDS Research, Pune, IND; 3 Pharmaceutical Medicine, Maharashtra University of Health Sciences, Nashik, IND; 4 Microbiology, Shri Bhausaheb Hire Government Medical College, Dhule, IND; 5 Internal Medicine, Shri Bhausaheb Hire Government Medical College, Dhule, IND; 6 Research, Maharashtra University of Health Sciences, Nashik, IND; 7 Dermatology, Venereology, and Leprology, Indian Council of Medical Research (ICMR) - National Institute of Translational Virology and AIDS Research, Pune, IND; 8 Paediatrics, Maharashtra University of Health Sciences, Nashik, IND

**Keywords:** covid-19, flow cytometry, interferon gamma release assay, sars-cov-2, t cell responses

## Abstract

Introduction

COVID-19 continues to have a global impact with the emergence of the latest variant of interest: JN.01, the commonest worldwide. While most studies on immune responses against SARS-CoV-2 are based on the evaluation of the humoral arm, data regarding cell-mediated immune responses are sparse and still emerging.

Materials and methods

A sample of 51 study participants was subjected to an interferon gamma release assay (QuantiFERON SARS-CoV-2). T cell (CD4+ or CD8+) responses to SARS-CoV-2 were characterized in a subset of 10 participants using multicolor flow cytometry.

Results

Thirty-seven participants had positive results for the anti-SARS-CoV-2 IgG antibody, and QuantiFERON SARS-CoV-2 assay antigen 1 and 2 positivity was observed in two participants only. In comparison, upon analyzing peripheral blood mononuclear cells using flow cytometry in a subset of 10 participants negative for the QuantiFERON SARS-CoV-2 assay, five had positive T cell immune responses.

Conclusion

Our study demonstrates higher positivity for T cell response among 10 adult individuals when tested using flow cytometry for peripheral blood mononuclear cell analysis as compared to the interferon gamma release assay. Such data on immune responses to SARS-CoV-2 will be useful in developing predictive B- and T cell-based immune correlates and algorithms for personalized risk assessment and clinical response evaluation.

## Introduction

As per the World Health Organization weekly epidemiological update on COVID-19, as of 10 November 2024, global cumulative confirmed cases were 7,76,841,264, with 36%, 27%, 25%, 8%, 3%, and 1% contributions from Europe, the Western Pacific, the Americas, South-East Asia, the Eastern Mediterranean, and Africa, respectively [1]. However, it is important to realize that these trends do not represent the actual figures since wastewater surveillance data suggest that clinical detection of COVID-19 cases underestimates the real burden from two- to 19-fold [1]. By the end of October 2024, JN.01 was reported to represent 99.5% of all circulating variants of SARS-CoV-2 worldwide, with KP.3.1.1 being the most prevalent JN.01 descendant [1,2].

In 2022, Moss, based on a review of several studies, reinforced the critical role of T cell response for protection against SARS-CoV-2 infection [3]. It has been observed that such virus-specific T cell responses lead to viral clearance, aid in infection prevention without seroconversion, and facilitate the development of cellular memory and viral variant recognition. Similar T cell responses are observed after vaccination. While most of the available literature regarding immune correlates of protection is based on the measurement of spike-specific antibody response or neutralizing titers, data on the measurement and profiling of cellular immune response are limited. With a continuous rise in the prevalence of confirmed cases, a concurrent COVID-19 vaccination drive, and disparate host responses, it is necessary to assess individual risk and clinical response using a “personalized approach” when conducting studies involving prospective cohorts, followed by the development of predictive B- and T cell-based immune correlates and algorithms [3].

## Materials and methods

Study design and population

The design for the study was exploratory and observational. The study sample included 51 participants above 18 years of age from the urban area of Malegaon city in Nashik District of Maharashtra, India, who had previously been tested for SARS-CoV-2 neutralizing antibody status in May 2022, and were tested with an interferon gamma release assay (IGRA; QuantiFERON SARS-CoV-2). Of those who tested negative for the SARS-CoV-2 neutralizing antibody and were willing to undergo additional specimen collection, a subset was subjected to flow cytometry analysis of peripheral blood mononuclear cells (PBMCs). This subset was limited to 10 participants due to resource constraints.

Specimen collection, transportation, and storage

Serum and whole blood specimens were collected in sterile and lithium heparin tubes (BD, USA) after obtaining informed consent in October 2022. Institutional ethical clearance was granted before initiation of the study (Maharashtra University of Health Sciences, Nashik, IEC No. MUHS/EC/30/2022).

SARS-CoV-2 serology

IgG antibody levels were detected with the COVID-19 Neutralizing Antibody Micro-ELISA Kit (J. Mitra & Co., India). Specimens with >30% inhibition on testing were labeled as antibody positive.

QuantiFERON SARS-CoV-2 (research use only) assay (QIAGEN, Germany)

This assay consisted of three antigen (Ag) tubes-namely, SARS-CoV-2 Ag1 (CD4+ epitopes derived from the S1 subunit of the spike protein), Ag2 (CD4+ and CD8+ epitopes from the S1 and S2 subunits of the spike protein), Ag3 (CD4+ and CD8+ epitopes from S1 and S2, plus immunodominant CD8+ epitopes derived from the whole genome)-and a Nil tube.

Flow cytometry analysis

**Figure 1:**
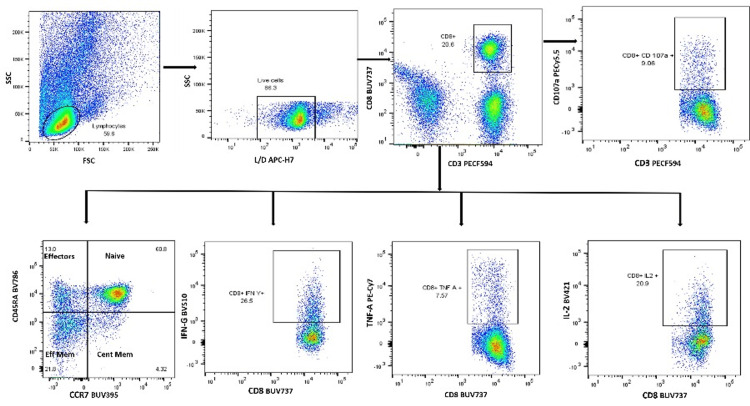
Gating strategy for CD4 and CD8 + T cell analysis using flow cytometry.

The T cell responses to SARS-CoV-2 were characterized in a subset of 10 vaccinated individuals with multicolor flow cytometry. The SARS-CoV-2-specific T cell response to SARS-CoV-2 peptide was assessed by intracellular cytokine staining. Briefly, the frozen PBMCs isolated from the participants were revived, rested overnight, and stimulated with SARS-CoV-2 peptides (spike PepTivator: pool of lyophilized peptides) in the presence of anti-CD107a (degranulation marker), followed by incubation with Brefeldin A and GolgiStop (monensin) at 37°C in 5% CO2. The next day, the cells were stained with a fluorescently labeled antibody cocktail (anti-CD3, anti-CD4, anti-CD8, anti-HLADR, anti-CD38, anti-CD45RA, and anti-CCR7). The cells were then washed, permeabilized, and stained with anti-IFN-γ, anti-TNF-α, and anti-IL-2 antibodies. Unstimulated PBMCs were used as a negative control, and those stimulated with staphylococcal enterotoxin B as a positive control. Cells were acquired on the FACSAria Fusion Flow Cytometer (BD Biosciences, United States) and analyzed using FlowJo software (Ashland, Oregon, United States). T cells from the stimulated PBMCs were differentiated based on CD4 and CD8 expression and assessed for activation status, memory status, and multiple cytokine (IFN-γ/IL-2/TNF-α) secretion. The Lymphocytes were identified by forward and side scatter. Lymphocytes were further drilled down to identify live cells. Live cells were then drilled down to gate CD3+ CD8(OR CD4+) T cells. CD8+ T cells were gated further to see expression of cytokines like CD107a, IL-2, TNFα and IFNγ and memory cells (Figure 1). The positivity criterion for a T cell response was a threshold of 1.0% after background subtraction.

Statistical analyses

Frequency distributions and descriptive statistics, such as measures of central tendency and dispersion, have been used to present the results of the study. The results were calculated with the help of statistical software SPSS Statistics for Windows, version 17.0 (SPSS Inc., Chicago, Ill, USA).

## Results

The study population consisted of 51 participants (27 male and 24 female) with a median age of 40 years (range: 18-69). Only one participant reported a history of past COVID-19 infection. Additionally, 48 participants mentioned that they have been vaccinated against COVID-19 at least once.

Table 1 shows the details of anti-SARS-CoV-2 IgG antibody and QuantiFERON SARS-CoV-2 assay Ag1 and Ag2 levels for the study participants (N = 51). Of the total sample, 37 had positive results for the anti-SARS-CoV-2 IgG antibody (>30%). In terms of detectable IFN-gamma secretion to Ag1 and Ag2, one and three participants had positive results, respectively.

**Table 1:** Details of anti-SARS-CoV-2 IgG antibody and QuantiFERON SARS-CoV-2 assay antigen 1 and 2 levels among the study participants (N = 51). * Antigen 1 and 2 values have been expressed after deduction of nil tube background values for each specimen; cut-off value < 0.15 IU/ml.

Particulars	Anti-SARS-CoV-2 IgG antibody (>30%)	Ag1*	Ag2*
No. of participants with positive results	37	1	3
Mean	48.20%	0.0248	0.0450

Details of study participants subjected to PBMC analysis using flow cytometry (n = 10) can be found in Table 2. All 10 participants had received the Covishield (Serum Institute of India Pvt. Ltd., Pune, India) vaccine, except for MMP 5, for whom the type of vaccine received is unknown. The age range of the participants was 22-69 years, and there were five male and five female participants. None of the participants had received a booster dose of the vaccine at the time of the study. Regarding the second dose, dates were known for three participants; for four, the status was “vaccine taken/date not known”; and we could not confirm the vaccine status for the remaining three.

**Table 2:** Details of study participants tested for peripheral blood mononuclear cell analysis by flow cytometry (n = 10).

Unique ID	Age	Gender	COVID-19 vaccination status	Type of vaccine	Date of first dose	Date of second dose	Booster dose
MMP 1	27	Male	Yes	Covishield	2 Nov 2021	Not known	No
MMP 2	38	Female	Yes	Covishield	9 Dec 2021	Not known	No
MMP 3	32	Female	Yes	Covishield	25 Nov 2021	Not known	No
MMP 4	62	Female	Yes	Covishield	10 Dec 2021	02 Feb 2022	No
MMP 5	47	Male	Yes	Details not known	17 Apr 2021	Not known	No
MMP 6	69	Female	Yes	Covishield	13 Sep 2021	17 Jan 2022	No
MMP 7	22	Male	Yes	Covishield	21 Oct 2021	Taken; date not known	No
MMP 8	33	Female	Yes	Covishield	27 Nov 2021	Taken; date not known	No
MMP 9	32	Male	Yes	Covishield	Taken; date not known	Taken; date not known	No
MMP 10	24	Male	Yes	Covishield	18 Oct 2021	13 Jan 2022	No

**Figure 2:**
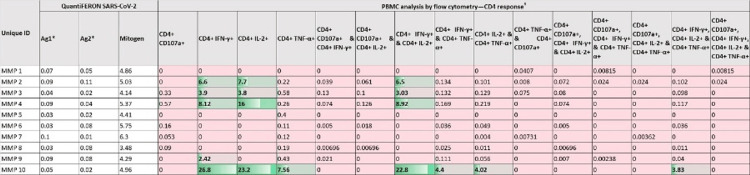
Heat map showing the distribution of QuantiFERON SARS-CoV-2 assay results and CD4+ responses measured by peripheral blood mononuclear cell analysis using flow cytometry. Shades of Green denote higher values/stronger CD4+ response using flow cytometry; Pink shade denotes lower values/weaker CD4+ response. # All flow cytometer data values are the final frequencies of cells stimulated with SARS-CoV-2 peptides after subtracting the values from unstimulated controls. Values above 1 should be considered positive. * Antigen 1 and 2 values have been expressed after deduction of nil tube background values for each specimen.

**Figure 3:**
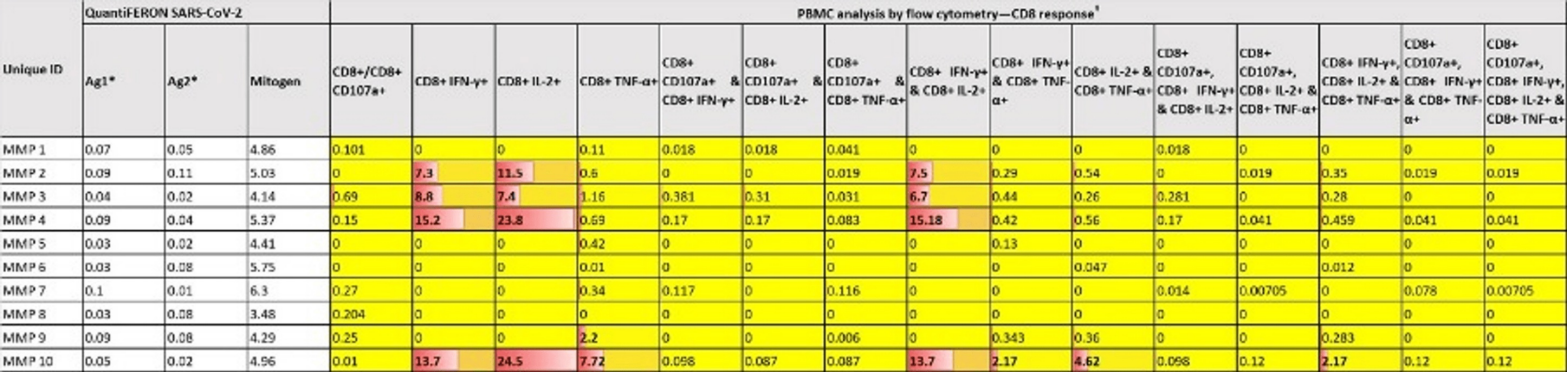
Heat map showing the distribution of QuantiFERON SARS-CoV-2 assay results and CD8+ responses measured by peripheral blood mononuclear cell analysis using flow cytometry. Shades of Orange denote higher values/stronger CD4+ response using flowcytometry; Yellow shade denote lower values/weaker CD4+ response. # All flow cytometer data values are the final frequencies of cells stimulated with SARS-CoV-2 peptides after subtracting the values from unstimulated controls. Values above 1 should be considered positive. * Antigen 1 and 2 values have been expressed after deduction of nil tube background values for each specimen.

Figure 2 and Figure 3 present participant-wise distribution of the QuantiFERON SARS-CoV-2 assay as well as PBMC analysis using flow cytometry results. The tables also indicate the CD4+ and CD8+ responses of the subset of 10 participants whose specimens were subjected to both tests. Five of these 10 participants (MMP 2, MMP 3, MMP 4, MMP 9, and MMP 10) exhibited cell-mediated immunity (CMI) response identified with the flow cytometer. In comparison, all 10 did not reveal any CMI response when analyzed with the QuantiFERON SARS-CoV-2 assay. SARS-CoV-2-specific CD4+ or CD8+ T cell responses were characterized by determining the frequencies of IFN-γ/IL-2/TNF-α secreting CD4+ and CD8+ T cells. IFN-γ- and IL-2-secreting CD4 and CD8 T cells were observed in 40% of the participants, whereas TNF-α-secreting cells were found to be rare. The frequencies of CD8+ T cells secreting both IFN-γ (median: 11.25%, IQR: 7.67%-14.83%) and IL-2 (median: 17.65%, IQR: 8.42%-24.33%) were higher than those of CD4+ T cells secreting IFN-γ (median: 6.60%, IQR: 3.16%-17.46%) and IL-2 (median: 11.85%, IQR: 4.77%-21.40%). Regarding SARS-CoV-2-specific CD4+ and CD8+ memory T cell response, central memory was observed in 50% and 30%, respectively, while it was 10% and 30%, respectively, for effector memory response.

## Discussion

Our study population included 51 participants with a median age of 40 years, with only one participant reporting a history of past COVID-19 infection. SARS-CoV-2-neutralizing antibodies were detectable in 48% of the sample, and 48 had been vaccinated against COVID-19. This study analyzed the immune responses of the participants to SARS-CoV-2 using various tests-the anti-SARS-CoV-2 IgG antibody and QuantiFERON SARS-CoV-2 assay, PBMC analysis, intracellular cytokine staining-and the frequencies of SARS-CoV-2-specific memory T cells. The results provide insight into the immune responses of the participants to SARS-CoV-2, which can be useful in developing predictive B- and T cell-based immune correlates and algorithms for personalized risk assessment and clinical response evaluation.

In a small feasibility study involving 16 participants who underwent QuantiFERON SARS-CoV-2 research use only (QFN SARS-CoV-2) IGRA (interferon-gamma release assay) and QIAreach Anti-SARS-CoV-2 total (anti-CoV-2) test, Jaganathan et al. reported that CD4+ and CD8+ T cell-mediated responses against SARS-CoV-2 were demonstrable in vaccinated subjects as well as in those who had recovered from natural infection [4]. The authors have highlighted that the significance of a T cell-mediated response to vaccination has recently gained greater recognition. The IGRA assay was suggested as an effective means for evaluating this response in vaccinated individuals. Stieber et al. studied a cohort of COVID-19-naive, healthy persons for up to 40 weeks by measuring T cell immune response after a two-dose vaccine regimen (mRNA-1273 (Moderna) or BNT162b2 (Pfizer-BioN) vaccine) using the QuantiFERON SARS-CoV-2 assay [5]. This longitudinal study reported a sustained T cell immune response over nine months, with no significant differences between the vaccines.

Fernández-González et al. assessed the clinical performance of a specific quantitative SARS-CoV-2 IGRA assay (Euroimmun, Germany) in 239 participants, comprising 152 convalescent, 54 vaccinated, and 33 uninfected and unvaccinated persons [6]. The study compared the presence of SARS-CoV-2-specific IgG, neutralizing antibodies, and IFN-γ responses in individuals who had recovered from COVID-19 and those who had been vaccinated. The IGRA method was found to modestly increase the detection of immunity overall, with a more pronounced contribution noted in convalescent patients with mild disease, where it increased the yield of serology by 13%. The authors indicated that IGRA is a consistent approach for assessing anti-SARS-CoV-2 T cell responses after either natural infection or vaccination. However, it is important to remember that these values for quantifying T cell response are dependent on disease severity and time lapse since the primary infection and/or vaccination.

In comparison, Aiello et al. reported a lower number of T cell immune responders with the QuantiFERON SARS-CoV-2 assay when compared to measurement by a homemade IGRA-SPIKE test [7]. Such discrepant results may have resulted from differences in the nature of the spike Ag used and the variance in concentrations in the two assays.

Dourdouna et al. studied humoral immunity and CMI using the QuantiFERON assay [8]. The 41 study subjects included unvaccinated convalescent children and adults, and vaccinated uninfected or vaccinated convalescent adults. All unvaccinated and a significant number of vaccinated participants had negative QuantiFERON assay results, possibly due to either a lowering of immunity or low sensitivity of this assay [8]. 

Johnson et al. demonstrated 100% sensitivity and specificity of QuantiFERON SARS-CoV-2 assay for detecting SARS-CoV-2 T cell responses in acute infection (12-21 days post positive PCR) [9]. The sensitivity to this test dropped to 12.5% in those with a history of past infection (172-444 days post-positive test). Thus, the QuantiFERON SARS-CoV-2 assay had a lower sensitivity for assessing long-term T cell responses [9].

Tormo et al. evaluated the performance of an in-house-developed flow cytometry assay for intracellular cytokine staining (FC-ICS) and the QuantiFERON SARS-CoV-2 assay (QF) for detection and quantification of T cell responses after COVID-19 vaccination [10]. A significant discordance was observed between the two methods, with the discrepant results mostly being FC-ICS positive/QF negative specimens, thereby suggesting greater sensitivity of the FC-ICS assay when compared to the QF assay [10]. 

In our study, participants were predominantly uninfected adults with a history of COVID-19 vaccination. We studied a subset of 10 participants who tested negative for SARS-CoV-2-neutralizing antibodies to evaluate T cell response, using both the QuantiFERON SARS-CoV-2 assay and PBMC analysis by flow cytometry. We found discrepant results, wherein all participants had negative results for the former test, while five exhibited a positive response when tested by the latter. Of the five participants with a positive flow cytometry result, four had a history of vaccination, while for one, vaccination status was unknown. Our data highlights the comparatively lower sensitivity of the QuantiFERON SARS-CoV-2 assay for measuring CMI response.

Our study has certain limitations. These include a small sample size of 10 study participants assessed for CMI using IGRA and flow cytometry concomitantly. There was heterogeneity among the participants regarding those with a history of natural infection, those vaccinated, or both. A control group of participants with no history of natural infection or vaccination was not assessed.

## Conclusions

The role of T cell responses is critical in determining the outcome of SARS-CoV-2 infection, viral clearance, disease severity, and long-term immunity. It is, therefore, important to monitor these responses to gauge immune protection levels in various population groups and identify those with weak T cell responses. Evaluation of cell-mediated immune responses will be essential in guiding future vaccination designs and strategies, especially regarding the development of variant-resistant immune protection.

While IGRA and flow cytometry are the two key methodologies available for measuring T cell responses, the latter has the advantage of providing granularity for a detailed analysis of cellular subsets. In the present study, flow cytometry-based PBMC analysis was able to detect CMI, in contrast to the QuantiFERON SARS-CoV-2 assay, among adults with a history of COVID-19 vaccination. IGRAs offer the advantages of being scalable and suitable for low-resource settings, but are associated with low sensitivity, especially for the measurement of long T cell responses to SARS-CoV-2 infection and vaccination. In comparison, flow cytometry-based PBMC analysis is more sensitive and quantifies both CD4+ and CD8+ subsets independently with detailed immune profiling and is better suited for research purposes. However, larger studies are essential to elaborate on cell-mediated immune response, along with providing a concurrent evaluation of the humoral immune response, so as to provide a composite picture of immune responses among the vaccinated population.
